# Current roles of endoscopy in the management of intraductal papillary mucinous neoplasm of the pancreas

**DOI:** 10.1111/den.12434

**Published:** 2015-02-05

**Authors:** Masao Tanaka

**Affiliations:** ^1^ Department of Surgery and OncologyGraduate School of Medical Sciences, Kyushu UniversityFukuokaJapan

**Keywords:** endoscopic retrograde cholangiopancreatography, endoscopic ultrasonography, epithelial subtype, intraductal papillary mucinous neoplasm, pancreatic cancer

## Abstract

Intraductal papillary mucinous neoplasm (IPMN) of the pancreas is characterized by intraductal papillary proliferation of mucin‐producing epithelial cells that exhibit various degrees of dysplasia. IPMN is classified into four histological subtypes (gastric, intestinal, pancreatobiliary, and oncocytic) according to its histomorphological and immunohistochemical characteristics. Endoscopic retrograde cholangiopancreatography plays a crucial role in the evaluation of these features of IPMN. Endoscopic ultrasonography (EUS) has proven to be more sensitive than computed tomography or magnetic resonance imaging for early detection of malignancy. The present review addresses the current roles of endoscopy and related techniques in the management of IPMN. The particular focus is on diagnosing IPMN and malignancy within IPMN, detecting pancreatic cancer concomitant with IPMN, differentiating the epithelial subtypes of IPMN, determining the optimal strategy for the management of branch duct IPMN, and discussing innovative endoscopic technology related to IPMN. The disadvantages of endoscopic examinations of IPMN and different attitudes toward EUS‐guided fine‐needle aspiration for IPMN between Japan (negative) and other countries (active) are also discussed.

## Introduction

Intraductal papillary mucinous neoplasm (IPMN) of the pancreas is a unique entity characterized by intraductal proliferation of mucinous epithelium that produces excessive mucin. The macroscopic morphology is divided into three types: main duct type (MD‐IPMN), branch duct type (BD‐IPMN), and mixed type.^1^ Endoscopy has played an important role in the diagnosis of IPMN since the discovery of this neoplasm. Ohashi *et al*.^2^, ^3^ first observed a patulous duodenal papilla that was markedly dilated by protruding mucin and reported it as the first case in 1980; they then reported a series of IPMN in 1981. This phenomenon was caused by MD‐IPMN and was considered to be a unique feature of ‘mucin‐hypersecreting pancreatic cancer’, which was considered a special type of pancreatic cancer associated with a better prognosis than that of ordinary pancreatic cancer.

The international consensus guidelines for the management of IPMN, as revised by the International Association of Pancreatology in 2012, do not recommend endoscopic retrograde cholangiopancreatography (ERCP) as a routine examination for sampling of fluid or brushings in IPMN. Thus, it is considered a special examination to be carried out only in the context of research.^4^ However, ERCP still has distinct roles in the investigation of IPMN. Moreover, endoscopic ultrasonography (EUS) has now become an indispensable modality with which to examine the pancreas harboring IPMN. This review describes the current roles of endoscopy, including ERCP and EUS, in the exploration of this unique and fascinating entity. Readers may refer to a recent publication for more details.^5^

## Diagnosis of IPMN

**Figure 1:**
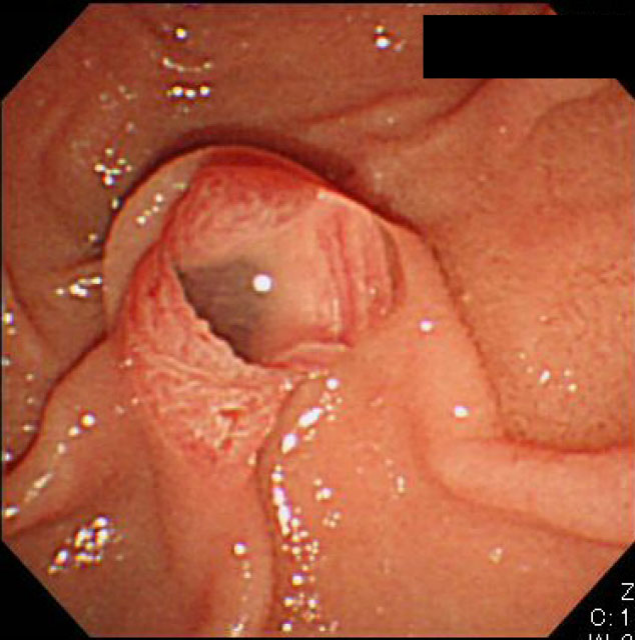
Patulous orifice of a dilated papilla with a protrusion of mucus, often seen in patients with intraductal papillary mucinous neoplasm (IPMN), particularly main duct‐type IPMN.

The threshold of the main pancreatic duct (MPD) diameter for suspicion of MD‐IPMN has been lowered to 5 mm to increase the sensitivity of the diagnosis without jeopardizing the specificity.^4^ ERCP is essential to confirm the presence of mucus in the MPD or at the papilla with or without the abnormal dilation of the papillary orifice (Fig. 1). This mucus is frequently seen in patients with MD‐IPMN. If these findings are absent, distinction of MD‐IPMN from chronic pancreatitis is very difficult, even by ERCP or EUS, and especially in patients with diffuse MPD dilation. In such cases, instead of carrying out immediate resection, it is necessary to observe the patient for a few months to determine whether the MPD dilation is progressive.

One of the specific features of BD‐IPMN is the presence of communication with the MPD, which is not a common feature of mucinous cystic neoplasm. However, the accumulation of mucus within the BD‐IPMN and its communicating branch frequently hampers the demonstration of the communication, even by ERCP. Instead, ERCP often reveals mucus moving within the MPD and changing its shape. The presence of mucus as shown by ERCP is regarded as a confirmatory finding of IPMN. EUS is often useful to demonstrate communication between the BD‐IPMN and MPD, even when occluded by mucus.

## Diagnosis of Malignancy in IPMN

Cytology is the gold standard examination technique for diagnosis of malignancy in IPMN. Cytological examination can be done using pancreatic juice obtained during ERCP or cyst fluid sampled by EUS‐guided fine‐needle aspiration (EUS‐FNA). Both of these approaches have advantages and disadvantages (Table 1).

**Table 1:** Advantages and disadvantages of endoscopy in the management of IPMN

	EUS	ERCP
Advantages		
Delineation of mural nodules	Clear	Not always possible
Demonstration of thickened septa	Clear	Seldom possible
Distinction of mural nodules from mucin	Possible by Doppler and/or contrast enhancement	Not clear
Diagnosis of malignancy	Possible by FNA cytology	Possible by pancreatic juice cytology
Detection of concomitant cancer	Possible when a mass is detectable	Possible by pancreatic juice cytology
Differentiation of epithelial subtype	Possible by FNA cytology	Possible by pancreatic juice cytology
Disadvantages		
Observer dependency	High	Minimal
Expertise required	Great	Great
Bleeding	Rare but possible	Extremely rare
Acute pancreatitis	Extremely rare	Rare but hampering its use in Western countries
Spillage and needle track seeding	Possible during FNA, hampering its use in Japan	No

ERCP, endoscopic retrograde cholangiopancreatography; EUS, endoscopic ultrasonography; FNA, fine‐needle aspiration; IPMN, intraductal papillary mucinous neoplasm.

Cytology of the pancreatic juice is associated with relatively low sensitivity of 10–50% for diagnosing malignancy (Table 2 ).^6^, ^7^, ^8^ The sensitivity can be increased to 80% with the use of repeated sampling through a nasopancreatic catheter.^9^ Irrigation of the pancreatic duct also reportedly increases the diagnostic sensitivity of ERCP cytology to 78%.^10^ Both histological grades and subtypes can be diagnosed fairly well by cell block cytology of the pancreatic juice, but these parameters are consistent with the histological findings of resected specimens in only 55.6% of BD‐IPMN. However, 100% accuracy can be obtained for MD‐IPMN and mixed‐type IPMN.^8^, ^11^, ^12^

**Table 2:** Reported data of pancreatic juice cytology in IPMN and pancreatic cancer

Author/Year	No. patients	Description
Yamaguchi *et al*., 2005^6^	71	Sensitivity for malignant IPMN of 14.1%, higher in main duct type
		Positivity in apparently benign IPMN may indicate presence of concomitant PDAC
Yamaguchi *et al*., 2005^7^	103	Diagnostic in all but one patient with IPMN and in 82.7% of patients with PDAC
Catheter	71	Sensitivity of 38.2% for IPMN
Pancreatoscopy	32	Sensitivity of 62.2% for IPMN, 25.4% for PDAC
Hibi *et al*., 2007^8^	19	79% consistent cytological and histological subclassifications
Mikata *et al*., 2013^9^	139	Data improved by ENPD (*P* = 0.0001)
Conventional	79	Sensitivity 39%, specificity 100%, PPV 100%, NPV 40%, accuracy 57%
ENPD for 3 days	60	Sensitivity 80%, specificity 100%, PPV 100%, NPV 71%, accuracy 87%
Sai *et al*., 2009^10^	24	Pancreatic duct lavage cytology in branch duct IPMN
		Sensitivity 78%, specificity 93%, PPV 88%, NPV 88%
Monzen *et al*., 2013^11^	23	Cell block cytology sufficient for typing/grading of IPMN in 20 patients, 95% of typing and 80% of grading consistent with histology
Hara *et al*., 2013^12^	36	Histological subtype accurately diagnosed in 42% of cases; rate improved to 89% with MUC staining (*P* < 0.01). HGD/invasive IPMN diagnosed with 77.2% sensitivity, 85.7% specificity, and 80.5% accuracy
Ohtsuka *et al*., 2014^49^	70	Accuracy 77%; 3 of 11 concomitant PDAC could be diagnosed only by pancreatic juice cytology

ENPD, endoscopic nasopancreatic drainage; HGD, high‐grade dysplasia; IPMN, intraductal papillary mucinous neoplasm; NPV, negative predictive value; PDAC, pancreatic ductal adenocarcinoma; PPV, positive predictive value.

Cyst fluid obtained by EUS‐FNA can also be used to diagnose malignant BD‐IPMN with a reported sensitivity and specificity of 67% and 88%, respectively, at a special institution with expertise in FNA and sophisticated cytodiagnostic interpretation.^13^ A higher sensitivity of 80% and a similar specificity of 85% were also reported from the same institution.^14^ Pitman *et al*.^15^ claimed the significance of ‘atypical epithelial cells’, not malignant cells, alone or in combination with a carcinoembryonic antigen (CEA) level of >2500 ng/mL as more accurate indicators of malignancy than are the Sendai criteria.

EUS provides several useful findings for the diagnosis of malignancy in IPMN. Mural nodules as the most significant sign of a high risk of malignancy can be clearly demonstrated by EUS. Hirono *et al*.^16^ analyzed 134 patients with resected BD‐IPMN and showed that a mural nodule size of >5 mm and a CEA level in the pancreatic juice of >30 ng/mL were independent predictors of malignancy. Ohno *et al*.^17^ reported contrast‐enhanced EUS findings in 87 patients with resected IPMN. Mural nodules, defined as blood flow‐supplied protrusions, were classified into four types: low papillary, polypoid, papillary, and invasive. The latter two types were diagnostic of malignancy with a sensitivity of 60.0%, specificity of 92.9%, and accuracy of 75.9%. The same group later reported the results of a long‐term retrospective study of 142 patients with BD‐IPMN.^18^ Malignant transformation occurred in nine patients (6.3%), with a 5‐year transformation rate of 10.7%. The existence of mural nodules at initial diagnosis and involvement of the MPD were significant predictors of malignant change of BD‐IPMN.

Although contrast‐enhanced power Doppler ultrasonography is associated with blooming artifacts, poor spatial resolution, low sensitivity to slow flow, and high sensitivity to motion artifacts, this new EUS system has further allowed for the observation of images of the pancreatic microcirculation and parenchymal perfusion without Doppler‐related artifacts.^19^ Contrast enhancement of mural nodules by computed tomography (CT) is described in the Fukuoka guidelines as a crucial finding suggestive of malignancy, but the same phenomenon by EUS should also be added. Thickening and irregularity of septa within BD‐IPMN can also be clearly visualized by EUS.

## Detection of Pancreatic Cancer Concomitant with IPMN

**Figure 2:**
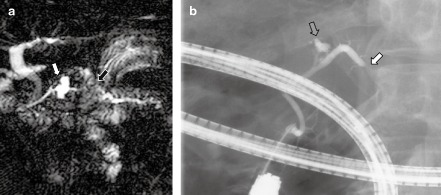
Concomitant pancreatic cancer found in a 71‐year‐old patient with branch duct‐type intraductal papillary mucinous neoplasm (BD‐IPMN). (a) Magnetic resonance cholangiopancreatography shows BD‐IPMN in the neck of the pancreas (closed arrow) and a stricture of the main pancreatic duct (MPD) in the body of the pancreas (open arrow) with upstream dilation. (b) Endoscopic retrograde cholangiopancreatography depicts complete obstruction of the MPD (closed arrow) separate from the BD‐IPMN, which is partly visualized (open arrow).

The development of ordinary pancreatic cancer in patients with IPMN has been drawing attention during the past two decades (Fig. 2). The first case of non‐invasive pancreatic cancer in a patient with BD‐IPMN was reported in 1997.^20^ The location of non‐invasive cancer was determined by endoscopic segmental cytology of the MPD with a balloon catheter. Since then, many series of patients have documented a 2–10% incidence of concomitant pancreatic cancer in patients with IPMN.^21^, ^22^, ^23^, ^24^, ^25^, ^26^, ^27^, ^28^, ^29^, ^30^ Uehara *et al*.^24^ reported that even ≤1‐cm BD‐IPMN were associated with an 8% risk of distinct pancreatic cancer during surveillance. The yearly incidence of distinct pancreatic cancer in patients with BD‐IPMN is reportedly 0.41–1.10%.^22^, ^23^, ^24^, ^25^, ^26^, ^27^, ^28^, ^29^, ^30^

More effective and efficient surveillance strategies for early detection of concomitant pancreatic cancer in patients with IPMN are needed. The intervals of the imaging surveillance proposed in the Fukuoka guidelines are now questioned because even surveillance done at 6‐month intervals may miss the diagnosis of pancreatic cancer at a sufficiently early stage.^31^, ^32^ Kamata *et al*.^33^ conducted EUS and other imaging examinations at 6‐month intervals in 102 patients with obviously benign BD‐IPMN and found distinct pancreatic cancer in seven patients (7%), but found no cases of malignant transformation of BD‐IPMN. Interestingly, CT and magnetic resonance imaging (MRI) carried out immediately after the detection of pancreatic cancer by EUS demonstrated the lesion in only 43% of cases. This suggests the crucial role of EUS, especially contrast‐enhanced harmonic EUS,^34^ in the early detection of pancreatic cancer despite its inherent weakness of observer dependency.

A few studies have investigated the characteristics of IPMN associated with distinct pancreatic cancer. Ideno *et al*.^35^ investigated the histological subtypes of IPMN in patients with concomitant pancreatic cancer and found a high frequency of the gastric subtype without *GNAS* mutation within codon 201. The epithelial subtypes of IPMN may be identified by ERCP cytology as described later and would help to predict the tendency of malignant transformation (most likely the intestinal type) and development of concomitant pancreatic cancer (mostly the gastric type).

Distinct pancreatic cancer may exist in the pancreatic segment to be left in place after pancreatectomy for IPMN or pancreatic cancer concomitant with IPMN. This can be examined by preoperative ERCP cytology^36^ or intraoperative irrigation cytology of the MPD.^37^ Moreover, pancreatic cancer may develop even after resection of IPMN. Ohtsuka *et al*.^38^ reported that 17 (9.9%) of 172 patients who underwent resection of IPMN subsequently developed distinct pancreatic cancer. If this condition is to be detected at a very early stage of development, patients who undergo IPMN resection by distal pancreatectomy should subsequently undergo ERCP cytology. The time interval between these cytological examinations should be determined in future studies.

Familial pancreatic cancer provides another opportunity for the early detection of pancreatic cancer.^39^ Patients with a strong family history of pancreatic cancer may develop multiple BD‐IPMN as well as pancreatic cancer during surveillance.^40^, ^41^, ^42^ The Fukuoka guidelines advocate the lower threshold for total pancreatectomy in such patients. However, because total pancreatectomy necessitates life‐long treatment of both pancreatogenic diabetes and exocrine deficit, ERCP cytology with or without balloon segmental cytology may help to decide whether to carry out pancreatic resection and, if done, determine the segment and extent of pancreatic resection before the uniform application of total pancreatectomy.^20^

## Differentiation of Epithelial Subtypes of IPMN

As reported by Furukawa *et al*.,^43^ IPMN is classified into four histological subtypes (gastric, intestinal, pancreatobiliary, and oncocytic) based on the histomorphological features of papillary proliferation and the immunohistochemical characteristics of mucin glycoproteins. Differentiation of these histological subtypes is achieved by postoperative histological examination of resected specimens of IPMN. Differentiation also has significance in predicting the prognosis, as previously described in several reports.^44^, ^45^, ^46^, ^47^ Colloid carcinoma derived from intestinal‐type IPMN and invasive oncocytic IPMN have far better prognoses,^45^ whereas tubular carcinoma derived from the gastric and pancreatobiliary types resembles ordinary pancreatic cancer.^46^, ^47^

In view of these observations, it is ideal to differentiate the subtypes preoperatively to consider the surgical indication and predict the prognosis. In a preliminary study by Hibi *et al*.,^8^ the cytological subclassification of pancreatic juice was consistent with the histological subtype of resected IPMN in 79% of cases. A subsequent study by Hara *et al*.^12^ explored the possibility of preoperatively determining IPMN histological subtypes in combination with mucin immunohistochemical cell staining of the pancreatic juice. The authors evaluated 36 patients who underwent preoperative pancreatic juice cytology and subsequent surgical resection of IPMN. Histological subtyping of cytological samples was consistent with the postoperative subtypes in 42% of patients without MUC staining and in 89% of patients with MUC staining (*P* < 0.01) and showed a significant correlation with the rate of malignancy. Preoperative pancreatic juice cytology with MUC staining may be useful for identification of the histological subtype of IPMN.

The above‐mentioned study also showed that the histological subtype is correlated with the *GNAS* mutational frequency.^48^ However, preoperative evaluation of the histological subtypes of IPMN is still in an investigational phase. Future studies involving mucin immunohistochemistry and analysis of specific genes may lead to the exploration of more definitive preoperative diagnoses of histological subtypes of IPMN. Endoscopic sampling of the pancreatic juice would likely play a crucial role in this context.

Cyst fluid obtained by EUS‐FNA may be another useful sample for histological subtype determination. However, differentiation of histological subtypes in FNA aspirates of IPMN has not yet been investigated.

## Disadvantages of Endoscopic Examination of IPMN

The disadvantages of endoscopic examination of IPMN are shown in Table 1. Acute pancreatitis remains a major disadvantage of ERCP cytology, as in other ERCP procedures, occurring in 10–14% of patients.^49^, ^50^, ^51^ Although most of cases of post‐ERCP acute pancreatitis shortly subside, up to 20% of patients may develop severe pancreatitis, which is sometimes fatal. Thus, great caution must be exercised when carrying out ERCP cytology.

Disadvantages of cyst fluid analysis for the diagnosis of malignant BD‐IPMN include acute pancreatitis, bleeding, and, although rare, gastric wall seeding and peritoneal dissemination of malignant cells. Anecdotal reports have described gastric wall invasion secondary to needle track seeding, or peritoneal dissemination as a result of leakage of cyst contents through needle holes.^52^, ^53^ A large cohort study compared 175 patients who underwent resection of IPMN after EUS‐FNA with 68 patients who underwent the resection without undergoing FNA.^54^ Although the study did not demonstrate a significant difference in the frequency of peritoneal seeding (2.3% *vs* 4.4%, respectively; *P* = 0.403), Japanese endosonographers refrain from EUS‐FNA in patients with possibly malignant BD‐IPMN because of concern regarding peritoneal dissemination and gastric wall seeding secondary to cyst fluid spillage and needle track implantation of malignant cells.

## Decision of Strategy for Management of BD‐IPMN

**Figure 3:**
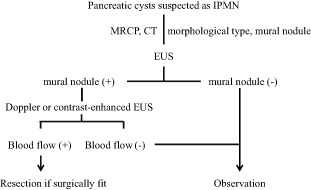
Algorithm of endoscopic management of intraductal papillary mucinous neoplasm (IPMN). CT, computed tomography; EUS, endoscopic ultrasonography; MRCP, magnetic resonance cholangiopancreatography.

Endoscopy plays a pivotal role in decision‐making in the management of BD‐IPMN. Whether to observe or operate on a BD‐IPMN depends on the presence and size of mural nodules within the BD‐IPMN (Fig. 3). Uehara *et al*.^55^ reported that a mural nodule of 10 mm was an appropriate indicator for immediate surgery. Mural nodules can be distinguished from mucus aggregates within the IPMN with the use of Doppler EUS and more definitively by contrast‐enhanced harmonic EUS to demonstrate the presence of a blood supply. Therefore, EUS is now essential in determining the optimal strategies with which to manage BD‐IPMN.

## Innovative Technology in Endoscopy

More recent innovations in modern technology have been applied to endoscopy and contribute to the management of IPMN. First, pancreatoscopy can allow for visualization of the papillary growths of IPMN in the MPD and help to accomplish targeted sampling of neoplastic cells for cytology and biopsy.^7^, ^56^, ^57^ Second, the ability of EUS‐elastography to provide supplementary information, mainly regarding pancreatic mass lesions, has also been investigated to distinguish mucinous (potentially malignant) from serous (mostly benign) cystic lesions by quantifying acoustic radiation force impulses from the cyst.^58^ Third, confocal laser endomicroscopy combined with EUS‐FNA can be used to enable real‐time microscopic and molecular imaging,^59^ demonstrating the presence of epithelial villous structures in cystic lesions of the pancreas, including IPMN.^60^ The utility, safety, and clinical significance of these new modalities need further evaluation.

## Conflict of Interests

Author declares no conflict of interests for this article.
